# Tocilizumab-effects on growth impairment in systemic juvenile idiopathic arthritis

**DOI:** 10.1186/1546-0096-9-S1-P130

**Published:** 2011-09-14

**Authors:** Takako Miyamae, Tomo Nozawa, Masako Kikuchi, Toshitaka Kizawa, Tomoyuki Imagawa, Shumpei Yokota

**Affiliations:** 1 Department of Pediatrics, Yokohama City University, Yokohama, Japan

## Background

Systemic juvenile idiopathic arthritis (s-JIA) is a subtype of chronic childhood arthritis of unknown etiology. The anti-IL-6 receptor monoclonal antibody, tocilizumab (TCZ), was developed, and we investigated the safety and efficacy of TCZ in children with this disorder.

## Aim

Growth analysis during the study was performed.

## Methods

Forty-five s-JIA patients (17 boys and 28 girls, 8.1±4.2 years of age, disease duration 4.1±3.2 years) who completed 144 visit (ca. 3 years) of phase-III study of TCZ were enrolled. The study had 6 week open-label phase (8mg/kh q2W) followed by 12 week randomized, double-blind withdrawal phase (8mg/kg q2w or placebo q2w).

Mean standard deviation score (SDS) for height, changes in SDS from baseline (⊿SDS), correlation between ⊿SDS and several factors such as age, gender, disease duration, corticosteroid dose exposure during the study, JIA core set response were evaluated. SDS for height calculated against the growth curve for Japanese population was used growth analysis.

## Results

Thirty-eight of 45 (84.4%) obtained clinical response at week 144. The mean baseline SDS-height was -2.67±1.97 and clearly showed growth impairment. Clear inverse correlation between baseline SDS-height and disease duration was recognized. There was no clear correlation between baseline corticosteroid dose and SDS-height. ⊿SDS at week 144 was 0.04±1.06, showing varied individual changes (⊿SDS:<-0.5 (n=10, mean 7.7 years) , ⊿SDS:-0.5≤, <0.5 (n=21, mean 8.1 years) ⊿SDS:0.5≤ (n=14, mean 8.2 years)). Patients with less corticosteroid exposure showed significant improvement in ⊿SDS compared with those with higher corticosteroid exposure during the study (average daily PSL equivalent dose <6.7 mg/body, (n=22) vs. ≥6.7 mg/body (n=23), *p*=0.001).

**Figure 1:**
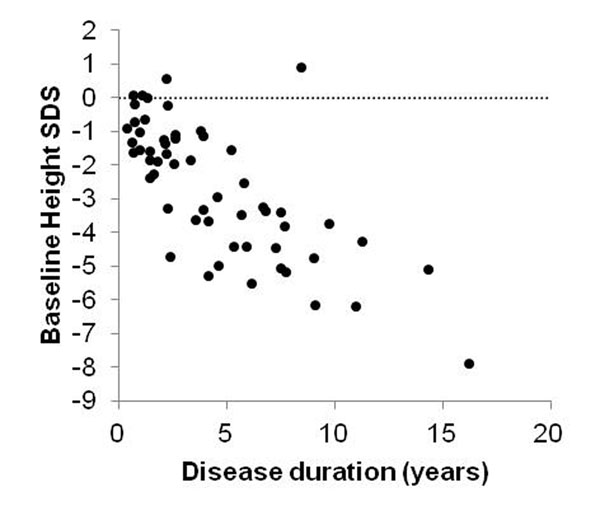


## Conclusion

Growth impairments evidenced by SDS-height were more prominent in patients with longer standing disease, which means importance of earlier intervention in the disease course. Catch up growth was observed in patients who required less or no corticosteroid during TCZ treatment for 3 years.

